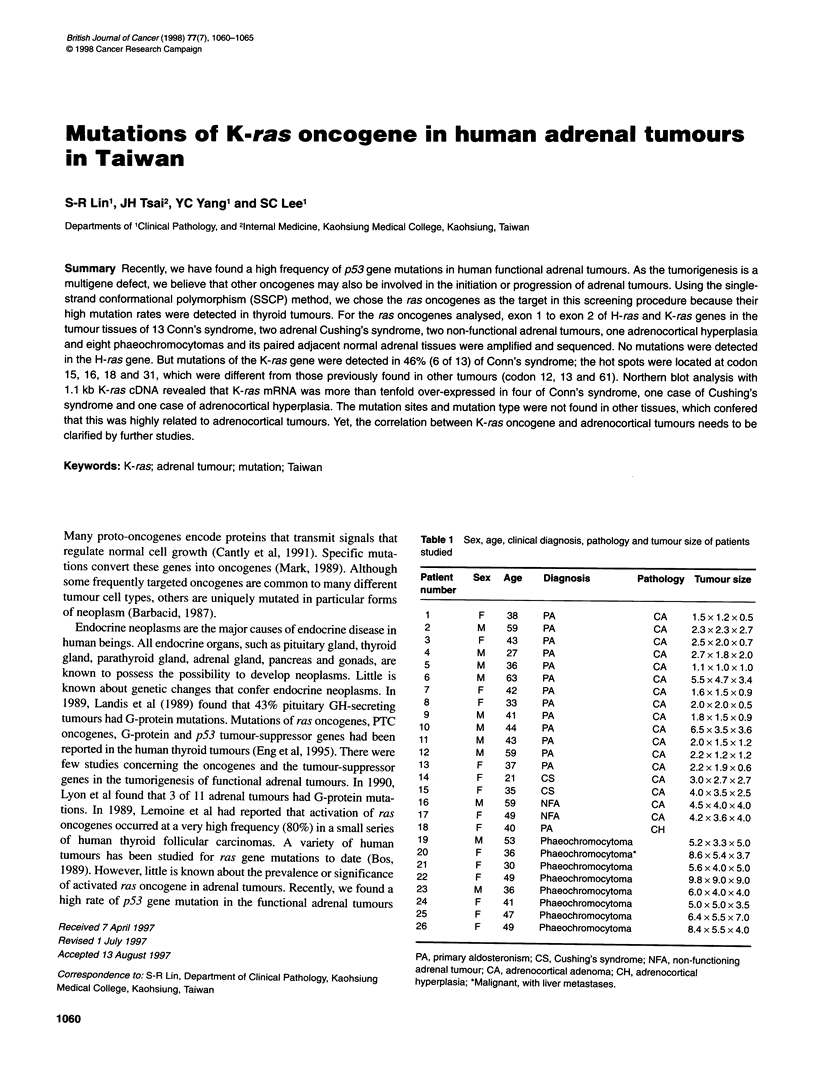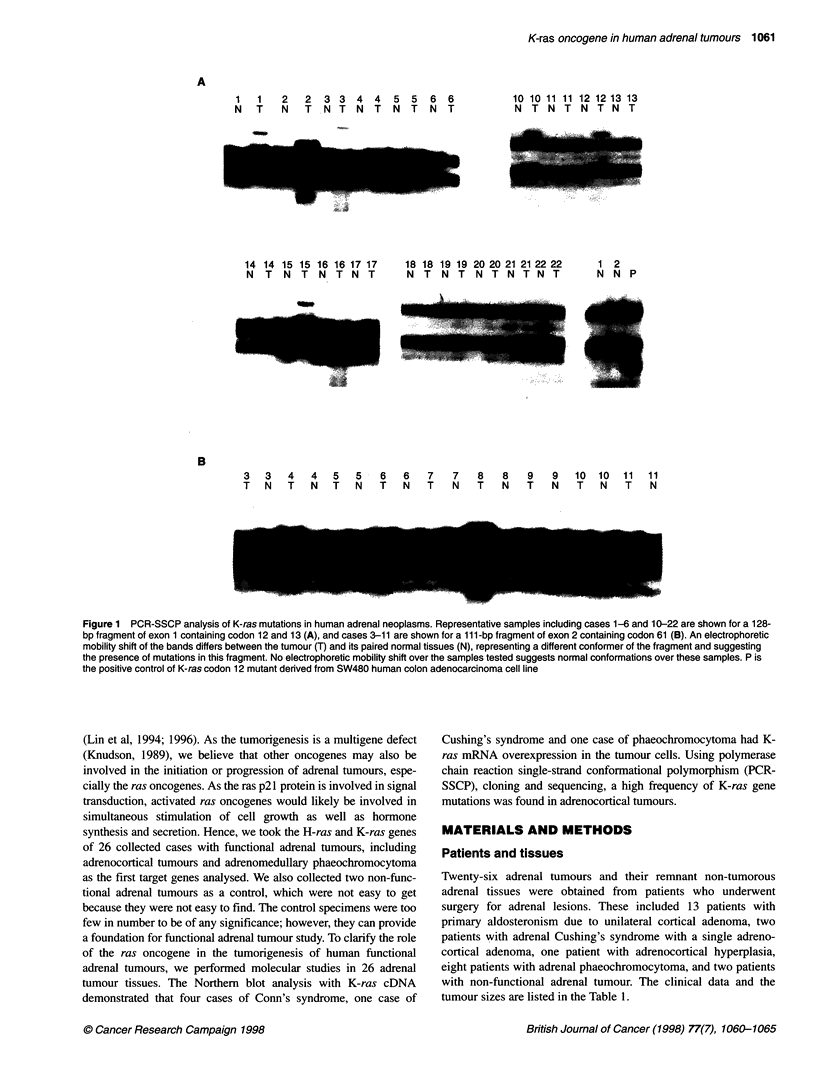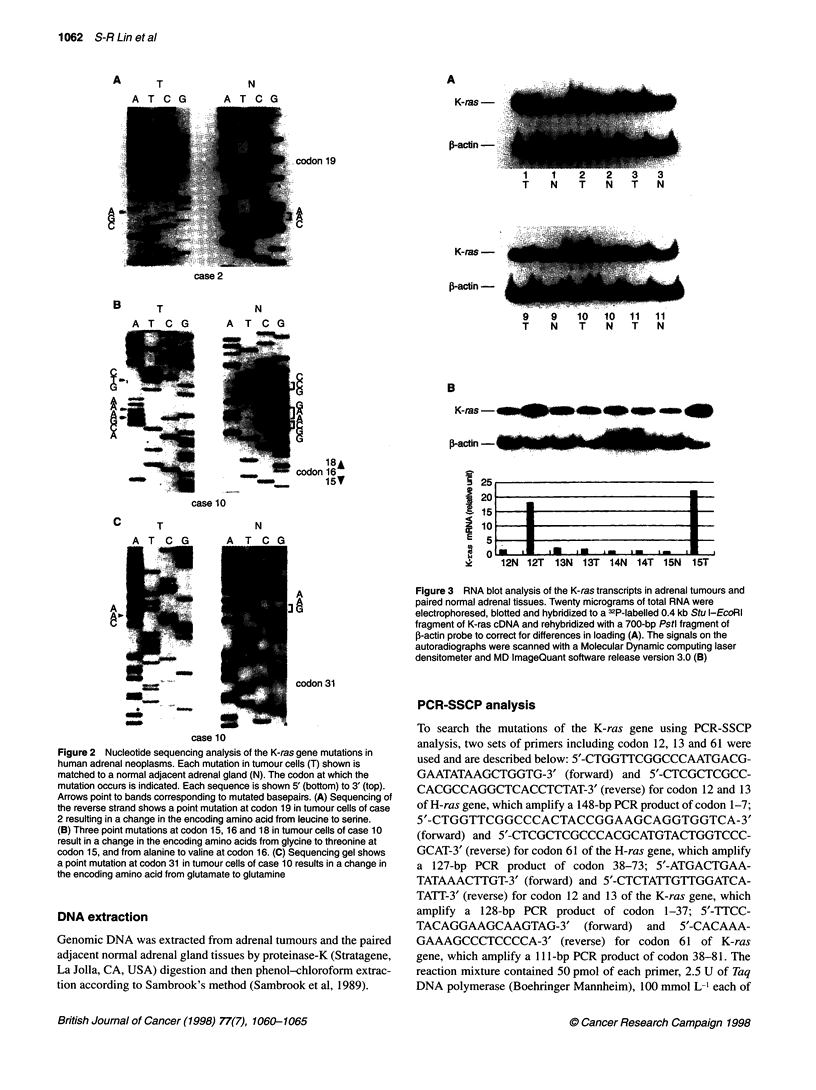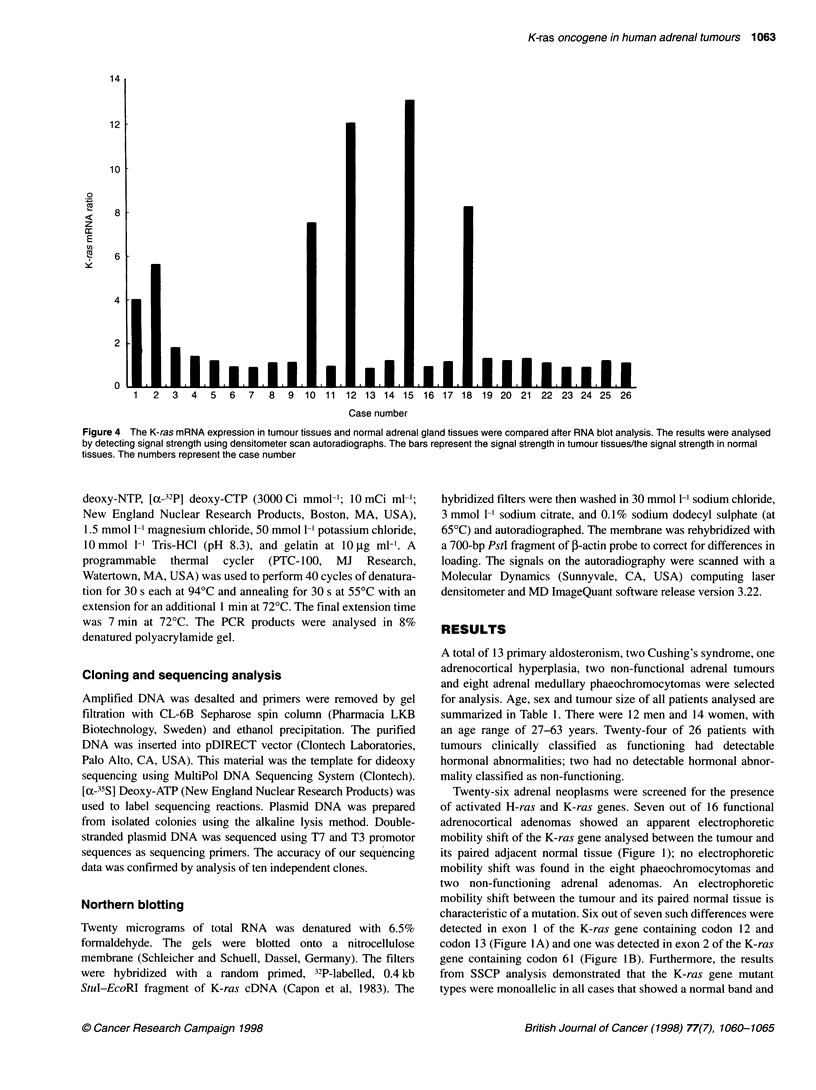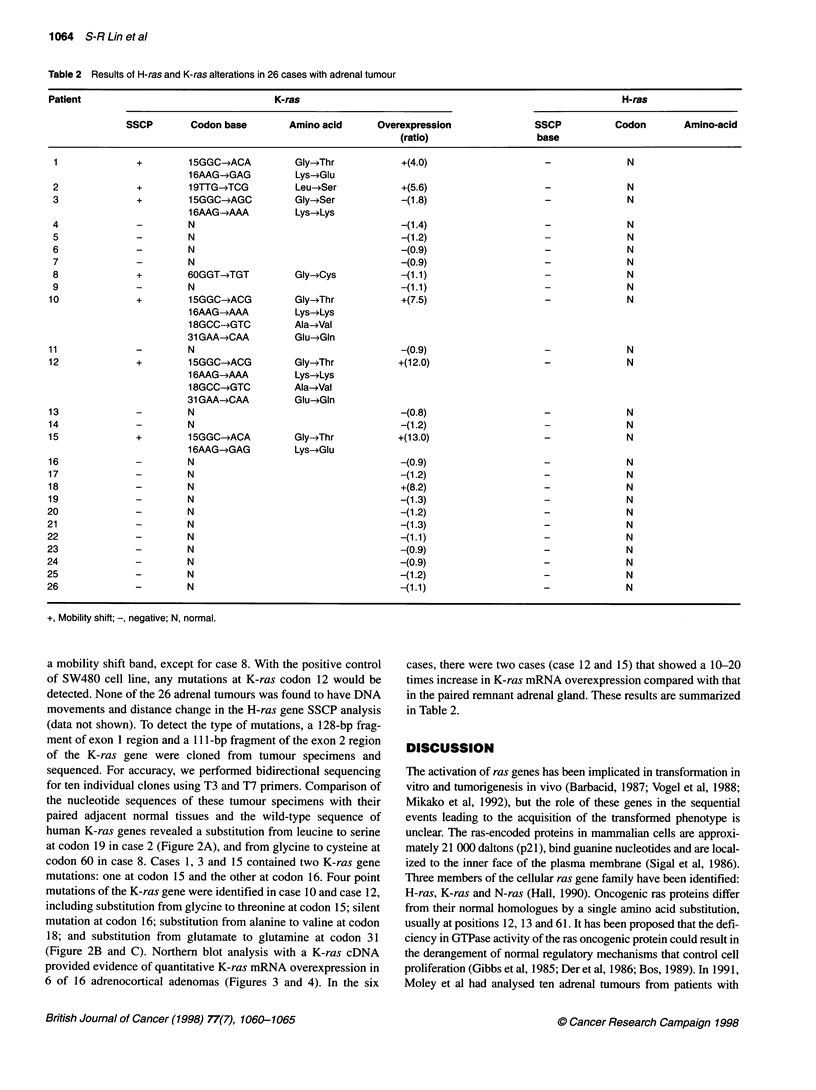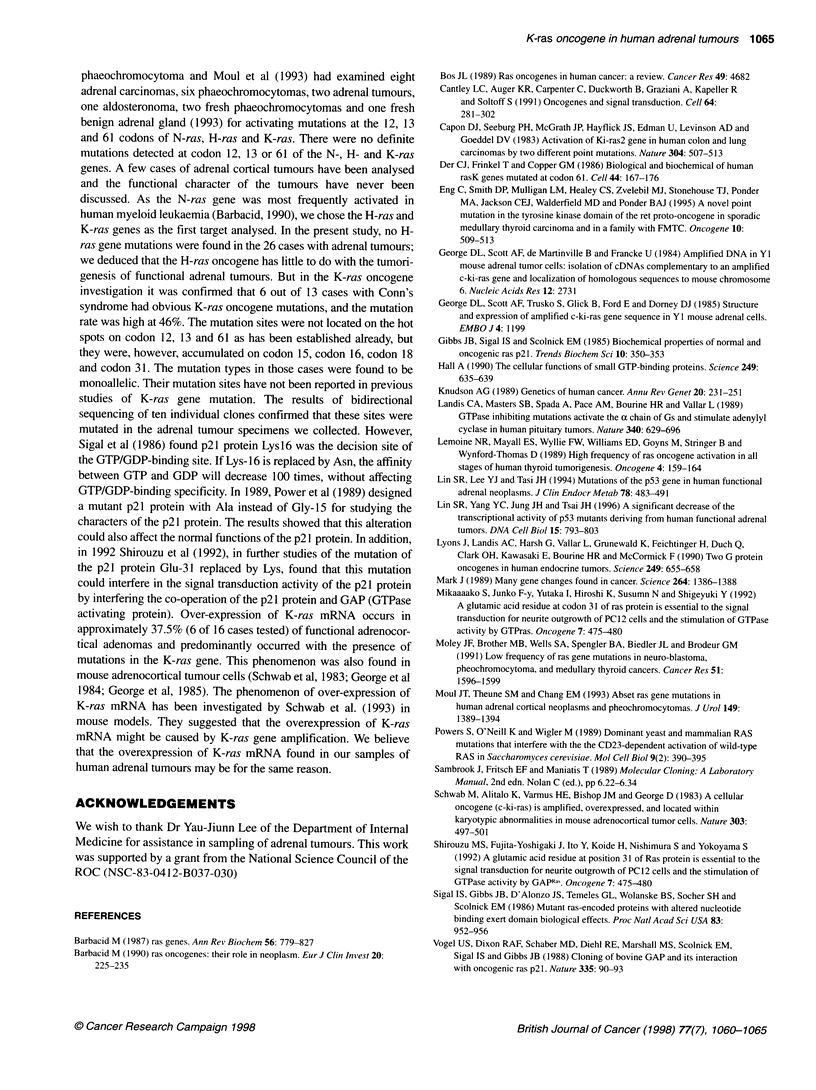# Mutations of K-ras oncogene in human adrenal tumours in Taiwan.

**DOI:** 10.1038/bjc.1998.177

**Published:** 1998-04

**Authors:** S. R. Lin, J. H. Tsai, Y. C. Yang, S. C. Lee

**Affiliations:** Department of Clinical Pathology, Kaohsiung Medical College, Taiwan.

## Abstract

**Images:**


British Journal of Cancer (1998) 77(7), 1060–1065
© 1998 Cancer Research Campaign

### Mutations of K-*ras* oncogene in human adrenal tumours in Taiwan

**S-R Lin[1], JH Tsai[2], YC Yang[1] and SC Lee[1]**

Departments of [1]Clinical Pathology, and [2]Internal Medicine, Kaohsiung Medical College, Kaohsiung, Taiwan

**Summary** Recently, we have found a high frequency of *p53* gene mutations in human functional adrenal tumours. As the tumorigenesis is a multigene defect, we believe that other oncogenes may also be involved in the initiation or progression of adrenal tumours. Using the single-strand conformational polymorphism (SSCP) method, we chose the *ras* oncogenes as the target in this screening procedure because their high mutation rates were detected in thyroid tumours. For the *ras* oncogenes analysed, exon 1 to exon 2 of H-*ras* and K-*ras* genes in the tumour tissues of 13 Conn's syndrome, two adrenal Cushing's syndrome, two non-functional adrenal tumours, one adrenocortical hyperplasia and eight phaeochromocytomas and its paired adjacent normal adrenal tissues were amplified and sequenced. No mutations were detected in the H-*ras* gene. But mutations of the K-*ras* gene were detected in 46% (6 of 13) of Conn's syndrome; the hot spots were located at codon 15, 16, 18 and 31, which were different from those previously found in other tumours (codon 12, 13 and 61). Northern blot analysis with 1.1 kb K-*ras* cDNA revealed that K-*ras* mRNA was more than tenfold over-expressed in four of Conn's syndrome, one case of Cushing's syndrome and one case of adrenocortical hyperplasia. The mutation sites and mutation type were not found in other tissues, which confered that this was highly related to adrenocortical tumours. Yet, the correlation between K-*ras* oncogene and adrenocortical tumours needs to be clarified by further studies.

**Keywords:** K-*ras*; adrenal tumour; mutation; Taiwan

Many proto-oncogenes encode proteins that transmit signals that regulate normal cell growth (Cantly et al, 1991). Specific mutations convert these genes into oncogenes (Mark, 1989). Although some frequently targeted oncogenes are common to many different tumour cell types, others are uniquely mutated in particular forms of neoplasm (Barbacid, 1987).

Endocrine neoplasms are the major causes of endocrine disease in human beings. All endocrine organs, such as pituitary gland, thyroid gland, parathyroid gland, adrenal gland, pancreas and gonads, are known to possess the possibility to develop neoplasms. Little is known about genetic changes that confer endocrine neoplasms. In 1989, Landis et al (1989) found that 43% pituitary GH-secreting tumours had G-protein mutations. Mutations of *ras* oncogenes, PTC oncogenes, G-protein and *p53* tumour-suppressor genes had been reported in the human thyroid tumours (Eng et al, 1995). There were few studies concerning the oncogenes and the tumour-suppressor genes in the tumorigenesis of functional adrenal tumours. In 1990, Lyon et al found that 3 of 11 adrenal tumours had G-protein mutations. In 1989, Lemoine et al had reported that activation of *ras* oncogenes occurred at a very high frequency (80%) in a small series of human thyroid follicular carcinomas. A variety of human tumours has been studied for *ras* gene mutations to date (Bos, 1989). However, little is known about the prevalence or significance of activated *ras* oncogene in adrenal tumours. Recently, we found a high rate of *p53* gene mutation in the functional adrenal tumours

*Received 7 April 1997*
*Revised 1 July 1997*
*Accepted 13 August 1997*

*Correspondence to:* S-R Lin, Department of Clinical Pathology, Kaohsiung Medical College, Kaohsiung, Taiwan

**Table 1** Sex, age, clinical diagnosis, pathology and tumour size of patients studied

| Patient number | Sex | Age | Diagnosis | Pathology | Tumour size |
|---|---|---|---|---|---|
| 1 | F | 38 | PA | CA | 1.5 × 1.2 × 0.5 |
| 2 | M | 59 | PA | CA | 2.3 × 2.3 × 2.7 |
| 3 | F | 43 | PA | CA | 2.5 × 2.0 × 0.7 |
| 4 | M | 27 | PA | CA | 2.7 × 1.8 × 2.0 |
| 5 | M | 36 | PA | CA | 1.1 × 1.0 × 1.0 |
| 6 | M | 63 | PA | CA | 5.5 × 4.7 × 3.4 |
| 7 | F | 42 | PA | CA | 1.6 × 1.5 × 0.9 |
| 8 | F | 33 | PA | CA | 2.0 × 2.0 × 0.5 |
| 9 | M | 41 | PA | CA | 1.8 × 1.5 × 0.9 |
| 10 | M | 44 | PA | CA | 6.5 × 3.5 × 3.6 |
| 11 | M | 43 | PA | CA | 2.0 × 1.5 × 1.2 |
| 12 | M | 59 | PA | CA | 2.2 × 1.2 × 1.2 |
| 13 | F | 37 | PA | CA | 2.2 × 1.9 × 0.6 |
| 14 | F | 21 | CS | CA | 3.0 × 2.7 × 2.7 |
| 15 | F | 35 | CS | CA | 4.0 × 3.5 × 2.5 |
| 16 | M | 59 | NFA | CA | 4.5 × 4.0 × 4.0 |
| 17 | F | 49 | NFA | CA | 4.2 × 3.6 × 4.0 |
| 18 | F | 40 | PA | CH | |
| 19 | M | 53 | Phaeochromocytoma | | 5.2 × 3.3 × 5.0 |
| 20 | F | 36 | Phaeochromocytoma* | | 8.6 × 5.4 × 3.7 |
| 21 | F | 30 | Phaeochromocytoma | | 5.6 × 4.0 × 5.0 |
| 22 | F | 49 | Phaeochromocytoma | | 9.8 × 9.0 × 9.0 |
| 23 | M | 36 | Phaeochromocytoma | | 6.0 × 4.0 × 4.0 |
| 24 | F | 41 | Phaeochromocytoma | | 5.0 × 5.0 × 3.5 |
| 25 | F | 47 | Phaeochromocytoma | | 6.4 × 5.5 × 7.0 |
| 26 | F | 49 | Phaeochromocytoma | | 8.4 × 5.5 × 4.0 |

PA, primary aldosteronism; CS, Cushing's syndrome; NFA, non-functioning adrenal tumour; CA, adrenocortical adenoma; CH, adrenocortical hyperplasia; *Malignant, with liver metastases.

**Figure 1** PCR-SSCP analysis of K-*ras* mutations in human adrenal neoplasms. Representative samples including cases 1–6 and 10–22 are shown for a 128-bp fragment of exon 1 containing codon 12 and 13 (**A**), and cases 3–11 are shown for a 111-bp fragment of exon 2 containing codon 61 (**B**). An electrophoretic mobility shift of the bands differs between the tumour (T) and its paired normal tissues (N), representing a different conformer of the fragment and suggesting the presence of mutations in this fragment. No electrophoretic mobility shift over the samples tested suggests normal conformations over these samples. P is the positive control of K-*ras* codon 12 mutant derived from SW480 human colon adenocarcinoma cell line

(Lin et al, 1994; 1996). As the tumorigenesis is a multigene defect (Knudson, 1989), we believe that other oncogenes may also be involved in the initiation or progression of adrenal tumours, especially the *ras* oncogenes. As the ras p21 protein is involved in signal transduction, activated *ras* oncogenes would likely be involved in simultaneous stimulation of cell growth as well as hormone synthesis and secretion. Hence, we took the H-*ras* and K-*ras* genes of 26 collected cases with functional adrenal tumours, including adrenocortical tumours and adrenomedullary phaeochromocytoma as the first target genes analysed. We also collected two non-functional adrenal tumours as a control, which were not easy to get because they were not easy to find. The control specimens were too few in number to be of any significance; however, they can provide a foundation for functional adrenal tumour study. To clarify the role of the *ras* oncogene in the tumorigenesis of human functional adrenal tumours, we performed molecular studies in 26 adrenal tumour tissues. The Northern blot analysis with K-*ras* cDNA demonstrated that four cases of Conn's syndrome, one case of Cushing's syndrome and one case of phaeochromocytoma had K-*ras* mRNA overexpression in the tumour cells. Using polymerase chain reaction single-strand conformational polymorphism (PCR-SSCP), cloning and sequencing, a high frequency of K-*ras* gene mutations was found in adrenocortical tumours.

### MATERIALS AND METHODS

### Patients and tissues

Twenty-six adrenal tumours and their remnant non-tumorous adrenal tissues were obtained from patients who underwent surgery for adrenal lesions. These included 13 patients with primary aldosteronism due to unilateral cortical adenoma, two patients with adrenal Cushing's syndrome with a single adrenocortical adenoma, one patient with adrenocortical hyperplasia, eight patients with adrenal phaeochromocytoma, and two patients with non-functional adrenal tumour. The clinical data and the tumour sizes are listed in the Table 1.

© Cancer Research Campaign 1998 

**Figure 2** Nucleotide sequencing analysis of the K-*ras* gene mutations in human adrenal neoplasms. Each mutation in tumour cells (T) shown is matched to a normal adjacent adrenal gland (N). The codon at which the mutation occurs is indicated. Each sequence is shown 5′ (bottom) to 3′ (top). Arrows point to bands corresponding to mutated basepairs. (**A**) Sequencing of the reverse strand shows a point mutation at codon 19 in tumour cells of case 2 resulting in a change in the encoding amino acid from leucine to serine. (**B**) Three point mutations at codon 15, 16 and 18 in tumour cells of case 10 result in a change in the encoding amino acids from glycine to threonine at codon 15, and from alanine to valine at codon 16. (**C**) Sequencing gel shows a point mutation at codon 31 in tumour cells of case 10 results in a change in the encoding amino acid from glutamate to glutamine

### DNA extraction

Genomic DNA was extracted from adrenal tumours and the paired adjacent normal adrenal gland tissues by proteinase-K (Stratagene, La Jolla, CA, USA) digestion and then phenol–chloroform extraction according to Sambrook's method (Sambrook et al, 1989).

**Figure 3** RNA blot analysis of the K-*ras* transcripts in adrenal tumours and paired normal adrenal tissues. Twenty micrograms of total RNA were electrophoresed, blotted and hybridized to a $^{32}$P-labelled 0.4 kb *Stu* I–*Eco*RI fragment of K-ras cDNA and rehybridized with a 700-bp *Pst*I fragment of β-actin probe to correct for differences in loading (**A**). The signals on the autoradiographs were scanned with a Molecular Dynamic computing laser densitometer and MD ImageQuant software release version 3.0 (**B**)

### PCR-SSCP analysis

To search the mutations of the K-*ras* gene using PCR-SSCP analysis, two sets of primers including codon 12, 13 and 61 were used and are described below: 5′-CTGGTTCGGCCCAATGACG-GAATATAAGCTGGTG-3′ (forward) and 5′-CTCGCTCGCC-CACGCCAGGCTCACCTCTAT-3′ (reverse) for codon 12 and 13 of H-*ras* gene, which amplify a 148-bp PCR product of codon 1–7; 5′-CTGGTTCGGCCCACTACCGGAAGCAGGTGGTCA-3′ (forward) and 5′-CTCGCTCGCCCACGCATGTACTGGTCCC-GCAT-3′ (reverse) for codon 61 of the H-*ras* gene, which amplify a 127-bp PCR product of codon 38–73; 5′-ATGACTGAA-TATAAACTTGT-3′ (forward) and 5′-CTCTATTGTTGGATCA-TATT-3′ (reverse) for codon 12 and 13 of the K-*ras* gene, which amplify a 128-bp PCR product of codon 1–37; 5′-TTCC-TACAGGAAGCAAGTAG-3′ (forward) and 5′-CACAAA-GAAAGCCCTCCCCA-3′ (reverse) for codon 61 of K-*ras* gene, which amplify a 111-bp PCR product of codon 38–81. The reaction mixture contained 50 pmol of each primer, 2.5 U of *Taq* DNA polymerase (Boehringer Mannheim), 100 mmol $L^{-1}$ each of

*© Cancer Research Campaign 1998*

**Figure 4** The K-*ras* mRNA expression in tumour tissues and normal adrenal gland tissues were compared after RNA blot analysis. The results were analysed by detecting signal strength using densitometer scan autoradiographs. The bars represent the signal strength in tumour tissues/the signal strength in normal tissues. The numbers represent the case number deoxy-NTP, [$\alpha$-$^{32}$P] deoxy-CTP (3000 Ci mmol$^{-1}$; 10 mCi ml$^{-1}$; New England Nuclear Research Products, Boston, MA, USA), 1.5 mmol l$^{-1}$ magnesium chloride, 50 mmol l$^{-1}$ potassium chloride, 10 mmol l$^{-1}$ Tris-HCl (pH 8.3), and gelatin at 10 $\mu$g ml$^{-1}$. A programmable thermal cycler (PTC-100, MJ Research, Watertown, MA, USA) was used to perform 40 cycles of denaturation for 30 s each at 94°C and annealing for 30 s at 55°C with an extension for an additional 1 min at 72°C. The final extension time was 7 min at 72°C. The PCR products were analysed in 8% denatured polyacrylamide gel.

### Cloning and sequencing analysis

Amplified DNA was desalted and primers were removed by gel filtration with CL-6B Sepharose spin column (Pharmacia LKB Biotechnology, Sweden) and ethanol precipitation. The purified DNA was inserted into pDIRECT vector (Clontech Laboratories, Palo Alto, CA, USA). This material was the template for dideoxy sequencing using MultiPol DNA Sequencing System (Clontech). [$\alpha$-$^{35}$S] Deoxy-ATP (New England Nuclear Research Products) was used to label sequencing reactions. Plasmid DNA was prepared from isolated colonies using the alkaline lysis method. Double-stranded plasmid DNA was sequenced using T7 and T3 promotor sequences as sequencing primers. The accuracy of our sequencing data was confirmed by analysis of ten independent clones.

### Northern blotting

Twenty micrograms of total RNA was denatured with 6.5% formaldehyde. The gels were blotted onto a nitrocellulose membrane (Schleicher and Schuell, Dassel, Germany). The filters were hybridized with a random primed, $^{32}$P-labelled, 0.4 kb *Stu*I–*Eco*RI fragment of K-*ras* cDNA (Capon et al, 1983). The hybridized filters were then washed in 30 mmol l$^{-1}$ sodium chloride, 3 mmol l$^{-1}$ sodium citrate, and 0.1% sodium dodecyl sulphate (at 65°C) and autoradiographed. The membrane was rehybridized with a 700-bp *Pst*I fragment of $\beta$-actin probe to correct for differences in loading. The signals on the autoradiography were scanned with a Molecular Dynamics (Sunnyvale, CA, USA) computing laser densitometer and MD ImageQuant software release version 3.22.

### RESULTS

A total of 13 primary aldosteronism, two Cushing's syndrome, one adrenocortical hyperplasia, two non-functional adrenal tumours and eight adrenal medullary phaeochromocytomas were selected for analysis. Age, sex and tumour size of all patients analysed are summarized in Table 1. There were 12 men and 14 women, with an age range of 27–63 years. Twenty-four of 26 patients with tumours clinically classified as functioning had detectable hormonal abnormalities; two had no detectable hormonal abnormality classified as non-functioning.

Twenty-six adrenal neoplasms were screened for the presence of activated H-*ras* and K-*ras* genes. Seven out of 16 functional adrenocortical adenomas showed an apparent electrophoretic mobility shift of the K-*ras* gene analysed between the tumour and its paired adjacent normal tissue (Figure 1); no electrophoretic mobility shift was found in the eight phaeochromocytomas and two non-functioning adrenal adenomas. An electrophoretic mobility shift between the tumour and its paired normal tissue is characteristic of a mutation. Six out of seven such differences were detected in exon 1 of the K-*ras* gene containing codon 12 and codon 13 (Figure 1A) and one was detected in exon 2 of the K-*ras* gene containing codon 61 (Figure 1B). Furthermore, the results from SSCP analysis demonstrated that the K-*ras* gene mutant types were monoallelic in all cases that showed a normal band and

© Cancer Research Campaign 1998

**Table 2** Results of H-*ras* and K-*ras* alterations in 26 cases with adrenal tumour

| Patient | K-*ras* | | | | H-*ras* | | |
|---|---|---|---|---|---|---|---|
| | SSCP | Codon base | Amino acid | Overexpression (ratio) | SSCP base | Codon | Amino-acid |
| 1 | + | 15GGC→ACA<br>16AAG→GAG | Gly→Thr<br>Lys→Glu | +(4.0) | – | N | |
| 2 | + | 19TTG→TCG | Leu→Ser | +(5.6) | – | N | |
| 3 | + | 15GGC→AGC<br>16AAG→AAA | Gly→Ser<br>Lys→Lys | –(1.8) | – | N | |
| 4 | – | N | | –(1.4) | – | N | |
| 5 | – | N | | –(1.2) | – | N | |
| 6 | – | N | | –(0.9) | – | N | |
| 7 | – | N | | –(0.9) | – | N | |
| 8 | + | 60GGT→TGT | Gly→Cys | –(1.1) | – | N | |
| 9 | – | N | | –(1.1) | – | N | |
| 10 | + | 15GGC→ACG<br>16AAG→AAA<br>18GCC→GTC<br>31GAA→CAA | Gly→Thr<br>Lys→Lys<br>Ala→Val<br>Glu→Gln | +(7.5) | – | N | |
| 11 | – | N | | –(0.9) | – | N | |
| 12 | + | 15GGC→ACG<br>16AAG→AAA<br>18GCC→GTC<br>31GAA→CAA | Gly→Thr<br>Lys→Lys<br>Ala→Val<br>Glu→Gln | +(12.0) | – | N | |
| 13 | – | N | | –(0.8) | – | N | |
| 14 | – | N | | –(1.2) | – | N | |
| 15 | + | 15GGC→ACA<br>16AAG→GAG | Gly→Thr<br>Lys→Glu | +(13.0) | – | N | |
| 16 | – | N | | –(0.9) | – | N | |
| 17 | – | N | | –(1.2) | – | N | |
| 18 | – | N | | +(8.2) | – | N | |
| 19 | – | N | | –(1.3) | – | N | |
| 20 | – | N | | –(1.2) | – | N | |
| 21 | – | N | | –(1.3) | – | N | |
| 22 | – | N | | –(1.1) | – | N | |
| 23 | – | N | | –(0.9) | – | N | |
| 24 | – | N | | –(0.9) | – | N | |
| 25 | – | N | | –(1.2) | – | N | |
| 26 | – | N | | –(1.1) | – | N | |

+, Mobility shift; –, negative; N, normal.

a mobility shift band, except for case 8. With the positive control of SW480 cell line, any mutations at K-*ras* codon 12 would be detected. None of the 26 adrenal tumours was found to have DNA movements and distance change in the H-*ras* gene SSCP analysis (data not shown). To detect the type of mutations, a 128-bp fragment of exon 1 region and a 111-bp fragment of the exon 2 region of the K-*ras* gene were cloned from tumour specimens and sequenced. For accuracy, we performed bidirectional sequencing for ten individual clones using T3 and T7 primers. Comparison of the nucleotide sequences of these tumour specimens with their paired adjacent normal tissues and the wild-type sequence of human K-*ras* genes revealed a substitution from leucine to serine at codon 19 in case 2 (Figure 2A), and from glycine to cysteine at codon 60 in case 8. Cases 1, 3 and 15 contained two K-*ras* gene mutations: one at codon 15 and the other at codon 16. Four point mutations of the K-*ras* gene were identified in case 10 and case 12, including substitution from glycine to threonine at codon 15; silent mutation at codon 16; substitution from alanine to valine at codon 18; and substitution from glutamate to glutamine at codon 31 (Figure 2B and C). Northern blot analysis with a K-*ras* cDNA provided evidence of quantitative K-*ras* mRNA overexpression in 6 of 16 adrenocortical adenomas (Figures 3 and 4). In the six cases, there were two cases (case 12 and 15) that showed a 10–20 times increase in K-*ras* mRNA overexpression compared with that in the paired remnant adrenal gland. These results are summarized in Table 2.

### DISCUSSION

The activation of *ras* genes has been implicated in transformation in vitro and tumorigenesis in vivo (Barbacid, 1987; Vogel et al, 1988; Mikako et al, 1992), but the role of these genes in the sequential events leading to the acquisition of the transformed phenotype is unclear. The ras-encoded proteins in mammalian cells are approximately 21 000 daltons (p21), bind guanine nucleotides and are localized to the inner face of the plasma membrane (Sigal et al, 1986). Three members of the cellular *ras* gene family have been identified: H-*ras*, K-*ras* and N-*ras* (Hall, 1990). Oncogenic ras proteins differ from their normal homologues by a single amino acid substitution, usually at positions 12, 13 and 61. It has been proposed that the deficiency in GTPase activity of the ras oncogenic protein could result in the derangement of normal regulatory mechanisms that control cell proliferation (Gibbs et al, 1985; Der et al, 1986; Bos, 1989). In 1991, Moley et al had analysed ten adrenal tumours from patients with

© Cancer Research Campaign 1998

phaeochromocytoma and Moul et al (1993) had examined eight adrenal carcinomas, six phaeochromocytomas, two adrenal tumours, one aldosteronoma, two fresh phaeochromocytomas and one fresh benign adrenal gland (1993) for activating mutations at the 12, 13 and 61 codons of N-*ras*, H-*ras* and K-*ras*. There were no definite mutations detected at codon 12, 13 or 61 of the N-, H- and K-*ras* genes. A few cases of adrenal cortical tumours have been analysed and the functional character of the tumours have never been discussed. As the N-*ras* gene was most frequently activated in human myeloid leukaemia (Barbacid, 1990), we chose the H-*ras* and K-*ras* genes as the first target analysed. In the present study, no H-*ras* gene mutations were found in the 26 cases with adrenal tumours; we deduced that the H-*ras* oncogene has little to do with the tumorigenesis of functional adrenal tumours. But in the K-*ras* oncogene investigation it was confirmed that 6 out of 13 cases with Conn's syndrome had obvious K-*ras* oncogene mutations, and the mutation rate was high at 46%. The mutation sites were not located on the hot spots on codon 12, 13 and 61 as has been established already, but they were, however, accumulated on codon 15, codon 16, codon 18 and codon 31. The mutation types in those cases were found to be monoallelic. Their mutation sites have not been reported in previous studies of K-*ras* gene mutation. The results of bidirectional sequencing of ten individual clones confirmed that these sites were mutated in the adrenal tumour specimens we collected. However, Sigal et al (1986) found p21 protein Lys16 was the decision site of the GTP/GDP-binding site. If Lys-16 is replaced by Asn, the affinity between GTP and GDP will decrease 100 times, without affecting GTP/GDP-binding specificity. In 1989, Power et al (1989) designed a mutant p21 protein with Ala instead of Gly-15 for studying the characters of the p21 protein. The results showed that this alteration could also affect the normal functions of the p21 protein. In addition, in 1992 Shirouzu et al (1992), in further studies of the mutation of the p21 protein Glu-31 replaced by Lys, found that this mutation could interfere in the signal transduction activity of the p21 protein by interfering the co-operation of the p21 protein and GAP (GTPase activating protein). Over-expression of K-*ras* mRNA occurs in approximately 37.5% (6 of 16 cases tested) of functional adrenocortical adenomas and predominantly occurred with the presence of mutations in the K-*ras* gene. This phenomenon was also found in mouse adrenocortical tumour cells (Schwab et al, 1983; George et al 1984; George et al, 1985). The phenomenon of over-expression of K-*ras* mRNA has been investigated by Schwab et al. (1993) in mouse models. They suggested that the overexpression of K-*ras* mRNA might be caused by K-*ras* gene amplification. We believe that the overexpression of K-*ras* mRNA found in our samples of human adrenal tumours may be for the same reason.

### ACKNOWLEDGEMENTS

We wish to thank Dr Yau-Jiunn Lee of the Department of Internal Medicine for assistance in sampling of adrenal tumours. This work was supported by a grant from the National Science Council of the ROC (NSC-83-0412-B037-030)

### REFERENCES

Barbacid M (1987) ras genes. *Ann Rev Biochem* **56**: 779–827

Barbacid M (1990) ras oncogenes: their role in neoplasm. *Eur J Clin Invest* **20**: 225–235

Bos JL (1989) Ras oncogenes in human cancer: a review. *Cancer Res* **49**: 4682

Cantley LC, Auger KR, Carpenter C, Duckworth B, Graziani A, Kapeller R and Soltoff S (1991) Oncogenes and signal transduction. *Cell* **64**: 281–302

Capon DJ, Seeburg PH, McGrath JP, Hayflick JS, Edman U, Levinson AD and Goeddel DV (1983) Activation of Ki-ras2 gene in human colon and lung carcinomas by two different point mutations. *Nature* **304**: 507–513

Der CJ, Frinkel T and Copper GM (1986) Biological and biochemical of human rasK genes mutated at codon 61. *Cell* **44**: 167–176

Eng C, Smith DP, Mulligan LM, Healey CS, Zvelebil MJ, Stonehouse TJ, Ponder MA, Jackson CEJ, Walderfield MD and Ponder BAJ (1995) A novel point mutation in the tyrosine kinase domain of the ret proto-oncogene in sporadic medullary thyroid carcinoma and in a family with FMTC. *Oncogene* **10**: 509–513

George DL, Scott AF, de Martinville B and Francke U (1984) Amplified DNA in Y1 mouse adrenal tumor cells: isolation of cDNAs complementary to an amplified c-ki-ras gene and localization of homologous sequences to mouse chromosome 6. *Nucleic Acids Res* **12**: 2731

George DL, Scott AF, Trusko S, Glick B, Ford E and Dorney DJ (1985) Structure and expression of amplified c-ki-ras gene sequence in Y1 mouse adrenal cells. *EMBO J* **4**: 1199

Gibbs JB, Sigal IS and Scolnick EM (1985) Biochemical properties of normal and oncogenic ras p21. *Trends Biochem Sci* **10**: 350–353

Hall A (1990) The cellular functions of small GTP-binding proteins. *Science* **249**: 635–639

Knudson AG (1989) Genetics of human cancer. *Annu Rev Genet* **20**: 231–251

Landis CA, Masters SB, Spada A, Pace AM, Bourine HR and Vallar L (1989) GTPase inhibiting mutations activate the α chain of Gs and stimulate adenylyl cyclase in human pituitary tumors. *Nature* **340**: 629–696

Lemoine NR, Mayall ES, Wyllie FW, Williams ED, Goyns M, Stringer B and Wynford-Thomas D (1989) High frequency of ras oncogene activation in all stages of human thyroid tumorigenesis. *Oncogene* **4**: 159–164

Lin SR, Lee YJ and Tasi JH (1994) Mutations of the p53 gene in human functional adrenal neoplasms. *J Clin Endocr Metab* **78**: 483–491

Lin SR, Yang YC, Jung JH and Tsai JH (1996) A significant decrease of the transcriptional activity of p53 mutants deriving from human functional adrenal tumors. *DNA Cell Biol* **15**: 793–803

Lyons J, Landis AC, Harsh G, Vallar L, Grunewald K, Feichtinger H, Duch Q, Clark OH, Kawasaki E, Bourine HR and McCormick F (1990) Two G protein oncogenes in human endocrine tumors. *Science* **249**: 655–658

Mark J (1989) Many gene changes found in cancer. *Science* **264**: 1386–1388

Mikaaaako S, Junko F-y, Yutaka I, Hiroshi K, Susumn N and Shigeyuki Y (1992) A glutamic acid residue at codon 31 of ras protein is essential to the signal transduction for neurite outgrowth of PC12 cells and the stimulation of GTPase activity by GTPras. *Oncogene* **7**: 475–480

Moley JF, Brother MB, Wells SA, Spengler BA, Biedler JL and Brodeur GM (1991) Low frequency of ras gene mutations in neuro-blastoma, pheochromocytoma, and medullary thyroid cancers. *Cancer Res* **51**: 1596–1599

Moul JT, Theune SM and Chang EM (1993) Abset ras gene mutations in human adrenal cortical neoplasms and pheochromocytomas. *J Urol* **149**: 1389–1394

Powers S, O'Neill K and Wigler M (1989) Dominant yeast and mammalian RAS mutations that interfere with the the CD23-dependent activation of wild-type RAS in *Saccharomyces cerevisiae*. *Mol Cell Biol* **9**(2): 390–395

Sambrook J, Fritsch EF and Maniatis T (1989) *Molecular Cloning: A Laboratory Manual*, 2nd edn. Nolan C (ed.), pp 6.22–6.34

Schwab M, Alitalo K, Varmus HE, Bishop JM and George D (1983) A cellular oncogene (c-ki-ras) is amplified, overexpressed, and located within karyotypic abnormalities in mouse adrenocortical tumor cells. *Nature* **303**: 497–501

Shirouzu MS, Fujita-Yoshigaki J, Ito Y, Koide H, Nishimura S and Yokoyama S (1992) A glutamic acid residue at position 31 of Ras protein is essential to the signal transduction for neurite outgrowth of PC12 cells and the stimulation of GTPase activity by $GAP^{Ras}$. *Oncogene* **7**: 475–480

Sigal IS, Gibbs JB, D'Alonzo JS, Temeles GL, Wolanske BS, Socher SH and Scolnick EM (1986) Mutant ras-encoded proteins with altered nucleotide binding exert domain biological effects. *Proc Natl Acad Sci USA* **83**: 952–956

Vogel US, Dixon RAF, Schaber MD, Diehl RE, Marshall MS, Scolnick EM, Sigal IS and Gibbs JB (1988) Cloning of bovine GAP and its interaction with oncogenic ras p21. *Nature* **335**: 90–93

© Cancer Research Campaign 1998